# Land use is associated with Indigenous maternal death occurrence through Native and Western food security

**DOI:** 10.1016/j.isci.2026.115948

**Published:** 2026-04-30

**Authors:** C.A. Ricci, A.E. Orloff, M. Wynne, L. Pflueger, P. Hallos, A. Lopez, M. Phelps, J.M. Postma, L.E. Hebert

**Affiliations:** 1 Washington State University, College of Nursing, 103 E Spokane Falls Blvd, Spokane, WA 99202, USA; 2 Washington State University, Institute for Research and Education to Advance Community Health (IREACH), 1100 Olive Wy #1200, Seattle, WA 9810, USA; 3 Washington State University, Elson. S. Floyd College of Medicine, 412 E Spokane Falls Blvd, Spokane, WA 99202, USA; 4 University of British Columbia, Institute of Resources, Environment and Sustainability, 2202 Main Mall, Vancouver, BC V6T 1Z4, Canada; 5 Spokane Tribal Network Tribal Food Sovereignty Initiative, 6230 Old School Rd, Wellpinit, WA 99040, USA; 6 Spokane Tribal Network Indigenous Birth Justice Initiative, 6230 Old School Rd, Wellpinit, WA 99040, USA; 7 Washington State University, Department of Animal Sciences, 1917 Marsh Rd, Yakima, WA 98901, USA; 8 Washington State University, School of Biological Sciences, PO Box 644236, Pullman, WA 99164-4236, USA; 9 Bureau of Reclamation, Yakima Basin, PO Box 646310, Pullman, WA 99164-6310, USA

**Keywords:** Public health, Nutrition, Food policy

## Abstract

Nearly every pregnancy-related American Indian/Alaska Native (AI/AN) maternal death within the United States is preventable. Factors contributing to AI/AN pregnancy disparities are numerous, complex, and interacting, but diet and nutritional disparities have major roles. AI/AN populations face pregnancy and food security disparities concurrently. While traditional (Native) foods can augment general food security, systematic practices like land use can impose access barriers. We used public data to investigate relationships between a main land use purpose (agriculture), Native and Western food securities, and AI/AN pregnancy-related maternal death. We also investigated land use (agriculture) associations with the nutritional quality of a vital traditional food for AI/AN Peoples from Northern Pacific latitudes (coho Pacific salmon, *Oncorhynchus kisutch*). We demonstrate that relationships exist between food security, land use, and AI/AN pregnancy. Relationships between land use and Native food security also indicate a need to consider the nutritional value of traditional foods consumed by pregnant AI/AN mothers.

## Highlights

• More agricultural land use showed lower food security and traditional food quality
• More agricultural land use showed more AI/AN maternal deaths
• High Native food security is potentially protective in AI/AN pregnancy
• A threshold for Native food security with potential policy relevance is identified

## Graphical abstract

**Figure:**
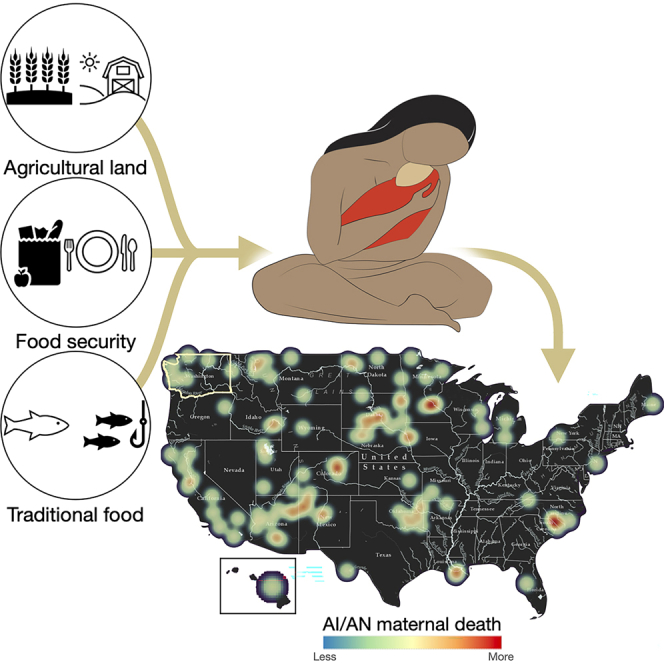

## Introduction

American Indian/Alaska Native (AI/AN) health disparities are severe, pervasive, and extend to pregnancy. Pregnancy health disparities are of particular concern because the United States is in a maternal death crisis. Annual nationwide estimates of maternal deaths from pregnancy and its complications (i.e., pregnancy-related maternal death) reached >700 in 2019 (20.1 deaths per 100,000 live births).^1^ This was prior to the overturning of *Roe* vs. *Wade* (i.e., the 2023 *Dobbs Decision*), which has prevented many pregnant people from receiving life-saving preventative and/or emergency care.^2^ State-level analyses indicate that AI/AN women experienced the greatest increases in maternal death over recent decades, with median state maternal mortality ratios rising from 14.0 deaths per 100,000 births in 1999 to 49.2 in 2019.^3^ Furthermore, retrospective analysis of AI/AN maternal deaths in the United States between 2017 and 2019 found that nearly every (>93%) pregnancy-related death was preventable^4^ (versus >84% in the general population^5^).

Factors contributing to AI/AN pregnancy disparities are numerous, complex, and interacting, but there is recognition that diet and nutrition disparities have major roles. For example, gestational diabetes is disproportionately prevalent among AI/AN populations (28.6 per 1,000 births in AI/AN populations compared to 8.7 per 1,000 births in non-Hispanic White populations in 2021^6^). Prevalences of several diet-related causes of death in AI/AN women of reproductive age (15–44 years) have also increased in the past two decades, including liver disease (+128.4%), cardiovascular disease (+55.3%), and diabetes mellitus (+213.0%).^7^ This has been concurrent with dietary shifts away from nutritionally healthy traditional foods and toward nutritionally deplete market foods.^8^ Broad patterns of dietary shifts toward market foods are characterized by purchases of more affordable foods that tend to be highly processed and high in sugar, salt, and unhealthy fat, while also being low in beneficial nutrients.^9^ These diets are most often a coping strategy for food insecurity, usually resulting from financial or geographic barriers to healthier options.^10^

Food security in academic literature typically applies a Western lens that follows the Food and Agriculture Organization of the United Nations (FAO) definition of “when all people, at all times, have physical and economic access to sufficient and safe nutritious food that meets their dietary needs and food preferences for an active and healthy life.”^11^ Likewise, research in the United States often follows United States Department of Agriculture (USDA) recommendations for measuring food security according to whether individuals have “adequate” access to quality, variety, and desirability of foods.^12^ However, many AI/AN and other Indigenous groups do not feel that these accurately reflect what food security encompasses for their Peoples.^13^ The First Nations Development Institute, for example, alternatively defines Native food security as, “when American Indians, Alaska Natives, and Native Hawaiians, at all times, have access to an abundance of culturally relevant foods to meet their dietary needs and preferences for a healthy tribal community.”^14^ This definition shifts the benchmarks for food security from sufficiency to abundance, and from individual needs to whole community thriving. It is furthermore underscored by inherent and inextricable relationships between traditional foods, ancestral lands, Tribal sovereignty, and Indigenous Peoples. We utilize this definition in the analyses presented in the current study.

Biological causal pathways that link nutrient deficits to health outcomes (including pregnancy outcomes) often focus on nutrient availability, absorption, and/or utilization.^15^ This can be modified by individual health behaviors to meaningful degrees in pregnancy. For example, in addition to maintaining a diversified diet^16^ and healthy levels of gestational weight gain,^17^ pregnant mothers can prioritize dietary intake of vitamin D,^18^^,^^19^ L-arginine, and L-citrulline^20^ to support fetal development. Increasing protein intake^21^ can also support maternal physical health, while fiber, magnesium, B vitamins, folate, niacin, vitamin D, and Vitamin E can support maternal mental health.^22^

Yet, it is vital to critically understand differences in individual abilities to enact health behaviors that achieve protective benefits. This is particularly relevant to AI/AN pregnancy, where historical and present-day violence has drastically changed the way AI/AN Peoples can live in relationship with their lands^23^^,^^24^ and has likewise resulted in significant declines in the availability of many traditional foods.^25^^,^^26^ This can, in turn, compound with food access hardships from other factors, such as living in remote subsistence villages or occupying a lower socioeconomic status. Many AI/AN individuals who are Western food insecure augment general food security with traditional foods, which can provide important nutrition.^27^ For example, Pacific salmon species (a vital traditional food for AI/AN and other Indigenous Peoples from Northern Pacific latitudes) historically made up approximately 60% of the Spokane Tribe diet^27^ (situated in the eastern region of what we now call Washington state) and are high in nutrients that are known to support healthy pregnancy. A well-studied example is docosahexaenoic acid (DHA), an omega-3 polyunsaturated fatty acid, or PUFA, that is abundant in fish and is essential for neonate brain development.^28^

Challenges accessing traditional foods exist. Commonly cited barriers include environmental degradation (e.g., pollution and climate change) and the privatization and/or destruction of ancestral lands.^29^ Economic factors such as cost of travel to ancestral hunting or harvesting areas (e.g., vehicle access, fuel, lodging, and time off work) are also cited as barriers to accessing traditional foods.^30^ On the other hand, major facilitators of Native food security include social and community factors like food sharing and traditional knowledge sharing/transfer programs.^30^ Some AI/AN leaders incorporate Indigenous and/or Tribal food sovereignty (I/TFS) principles into these programs as potential solutions to hunger and food insecurity in their communities. Indigenous Food Sovereignty can have different meanings between Indigenous groups but is generally regarded as a sacred responsibility held by Indigenous Peoples to participate in traditional practices that cultivate healthy and interdependent relationships with land, sea, air, soil, plants, and animals; and the cultural and traditional foods which are produced though these relationships are in turn viewed as sacred.^31^ Similarly, TFS has been invoked to describe how Indigenous food sovereignty concepts are achieved in individual communities to revitalize and honor a Nation’s specific cultural values and ancestral lifeways.^32^^,^^33^

**Figure 1:**
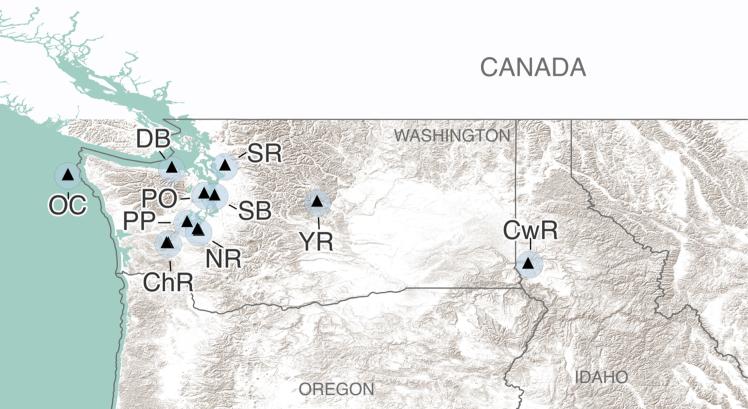
Catch locations for coho Pacific salmon samples Exact catch locations are represented by black triangles; light blue circles surrounding catch locations are 15 mi buffer zones. ChR: Chehalis River; CwR: Clearwater River, DB: Dungeness Bay, NR: Nisqually River, OC: outer coast, PP: Peale Passage, PO: Port Orchard, SB: Salmon Bay Waterway, SR: Stillaguamish River, YR: Yakima River.

We asked if land use had the potential to influence AI/AN maternal health through food security and used agriculture as a representative land use variable (agriculture is the number one land use purpose within the United States territories, comprising >1/2 of the total United States land area as of 2026^34^). We first tested the nationwide distributions of agriculture for associations with Native and Western food security, and the occurrence of AI/AN pregnancy-related maternal death. Then, as a case study, we investigated associations between nutritional characteristics of a traditional food (coho Pacific salmon, *Oncorhynchus kisutch*) sampled across a Pacific Northwest region (primarily Washington, United States, Figure 1). Nutritional quality was tested because changes to traditional foods that are available can have specific consequences for the Native food security of AI/AN Peoples, including mothers. We acknowledge that poor pregnancy outcomes beyond maternal death occur (including long-term and severe maternal morbidity), and that poor pregnancy outcomes can additionally impact offspring health and development. AI/AN pregnancy-related maternal death was investigated here because of the United States maternal death crisis.

## Results

### Occurrence of AI/AN pregnancy-related maternal death can be partly explained by relationships between acreage dedicated to large agricultural operations, impacts on native food security, and generalized Western food insecurity

**Figure 2:**
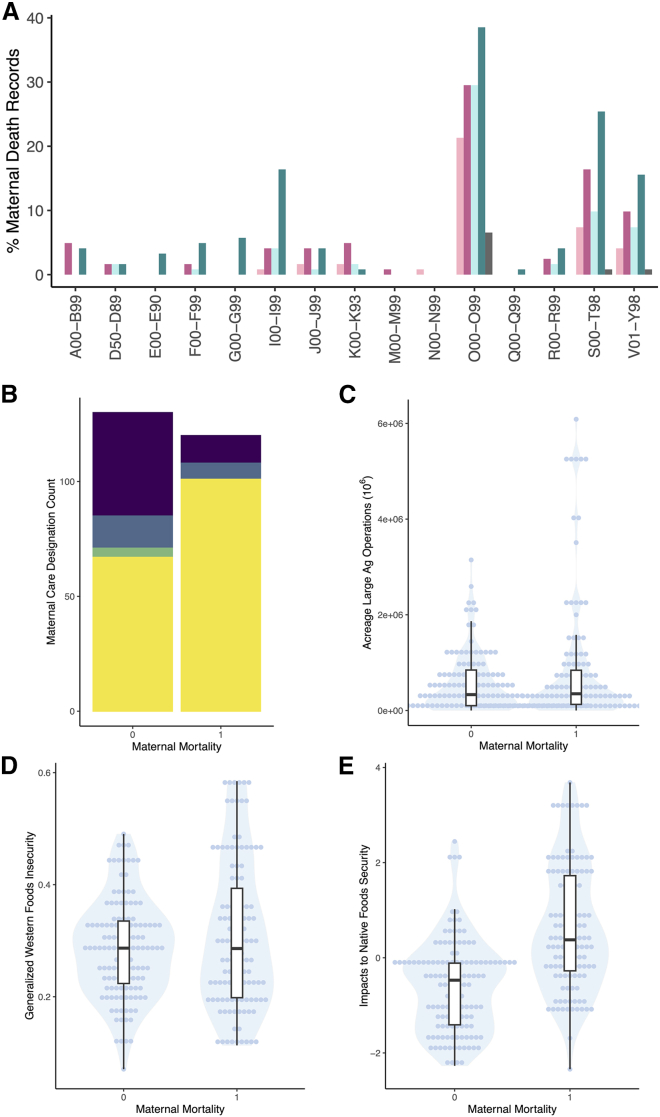
Causes of maternal death and univariate comparisons of non-observation (0) and observation (1) counties (A) Frequency of primary and contributing causes of AI/AN pregnancy-related maternal death during pregnancy and in the postpartum. Pink shades and teal shades represent the frequency of causes cited for maternal death during pregnancy and in the postpartum, respectively. Lighter shades represent the frequency of citations as a primary cause of death. Darker shades represent the frequency of citations as a contributing cause of death. Gray bars represent records with missing pregnancy status data. Frequencies for causes of death are aggregated and categorized under ICD10 chapters. A00-B99: chapter I. Certain infectious and parasitic diseases; D50-D89: chapter III. Diseases of the blood and blood-forming organs and certain disorders involving the immune mechanism; E00-E90: chapter IV. Endocrine, nutritional and metabolic diseases; F00-F99: chapter V. Mental and behavioral disorders; G00-G99: chapter IV. Diseases of the nervous system; I00-I99: chapter IX. Diseases of the circulatory system; J00-J99: chapter X. Diseases of the respiratory system; K00-K93: chapter XI. Diseases of the digestive system; M00-M99: chapter XIII. Diseases of the musculoskeletal system and connective tissue; N00-N99: chapter XIV. Diseases of the genitourinary system; O00-O99: chapter XV. Pregnancy, childbirth, and the puerperium; Q00-Q99: chapter XVII. Congenital malformations, deformations and chromosomal abnormalities; R00-R99: chapter XVIII. Symptoms, signs, and abnormal clinical and laboratory findings, not elsewhere classified; S00-T98: chapter XIX. Injury, poisoning, and certain other consequences of external causes; V01-Y98; chapter XX. External causes of morbidity and mortality. Data are counts of records expressed as a percentage of total records included in our final dataset. (B) Stacked bar plot of county maternal care access rating. The height of the bar represents the count of counties included in the analysis that are rated respectively. Yellow: highest rating of maternal care access; green: moderate maternal care access; blue: low maternal care access; purple: maternal care desert. (C) Violin plot of acreage dedicated to large agricultural operations. (D) Violin plot of generalized Western food insecurity. (E) Violin plot of Native food security impacts. Boxplots superimposed on violin plots (C-E) represent the distribution and the interquartile range of obseervation and non-observation counties.

A total of 128 unique causes of death were present in AI/AN pregnancy-related maternal death records identified in data years 2016–2019, and 2022. The causes of death were grouped into 15 distinct categories that corresponded to chapters in the International Classification of Diseases, 10th Revision (ICD10) under which the cause was classified. Causes of death are reported at the chapter level to protect potentially identifiable information. For both the pregnancy and postpartum phases, we summarized the frequencies with which causes of death were cited as a primary or contributing cause (Figure 2A). 4 categories reached citations in ≥10% of AI/AN pregnancy-related maternal death records (I00-I99: diseases of the circulatory system; O00-O99: pregnancy, childbirth, and the puerperium; S00-T98: injury, poisoning, and certain other consequences of external causes; V01-Y98: external causes of morbidity and mortality; Figure 2A). Approximately 85% of causes classified under I00-I99 were related to heart disease (data not shown) and were most frequently cited as contributing causes during the postpartum phase. Mental health causes of death were major portions of S00-T98 and V01-Y98 (approximately 56% and 85%, respectively; data not shown). O00-O99 causes of death did not have a unifying theme, although several diet-related causes were cited (e.g., hypertension and diabetes mellitus). In all 4 categories, causes of death were cited with greater frequency if mothers died during the postpartum phase. This reflected the overall greater number of postpartum deaths in our dataset (70 postpartum deaths versus 46 deaths while pregnant; 6 records lacked pregnancy status data; data not shown).

We compared 11 generalized linear models (Table 1) to identify the most informative combination of our variables of interest (selected *a priori*). The model using county-level acreage dedicated to large agricultural operations, Native food security impacts, and generalized Western food insecurity explained the occurrence of AI/AN pregnancy-related maternal death best (Model J, Tjur’s R^2^ = 0.292, RMSE = 0.429, PCP = 0.646, Table 1). Generally, Tjur’s R^2^ for univariate models did not exceed 0.02, with the exception of Native food security impacts, which nearly reached 0.2 (Table 1). County-level maternal care access did not explain AI/AN pregnancy-related maternal death occurrence. The majority of counties rated highest for maternal care access (∼60%) were associated with the occurrence of AI/AN pregnancy-related maternal death, while the majority of counties classified as maternal care deserts (∼79%) were those where AI/AN pregnancy-related maternal death was not observed (Figure 2B). Standalone variables included in the most explanatory model were also not individually informative to the occurrence of AI/AN pregnancy-related maternal death (Figures 2C–2E).

**Table 1:** Model comparisons

Model	Variables	Tjur’s R^2^	RMSE	PCP
J	Native food, Western food (gen), ag acreage	0.292	0.429	0.646
K	Native food, Western food (AI/AN), ag acreage	0.250	0.436	0.625
E	Native food, Westernfood (gen)	0.226	0.438	0.613
F	Native food, Westernfood (AI/AN)	0.195	0.441	0.597
C	Native food	0.194	0.441	0.597
I	Native food, ag acreage	0.190	0.445	0.595
H	Western food (AI/AN), ag acreage	0.057	0.485	0.528
G	Western food (gen), ag acreage	0.038	0.494	0.518
A	Western food (gen)	0.013	0.498	0.506
B	Western food (AI/AN)	0.008	0.498	0.504
D	Ag acreage	0.001	0.500	0.500

Native food: county Native food security impacts; Western food (gen): county generalized Western food insecurity; ag acreage: county acreage dedicated to large agricultural operations; RMSE: root mean squared error; PCP: % correct predicted. Model goodness-of-fit comparisons for county-level factors associated with AI/AN pregnancy-related maternal death occurrence. Models arranged in descending order from best fit to worst fit.

**Figure 3:**
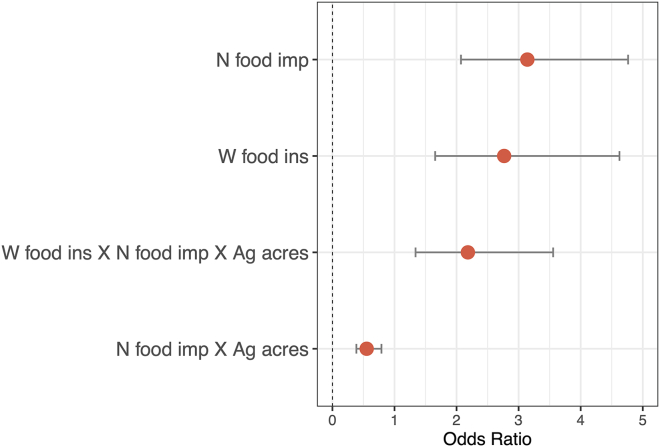
Model J odds ratio plot Odds ratio plot for the effect size of variables included in the most informative model that contribute to AI/AN pregnancy-related maternal death occurrence. Variables not reaching the Bonferroni multiple comparisons significance threshold (*p* > 0.004) are not shown. Error bars represent the 97.5% confidence interval. N food imp: county Native food security impacts; W food ins: county generalized Western food insecurity; Ag acres: county acreage dedicated to large agricultural operations.

**Figure 4:**
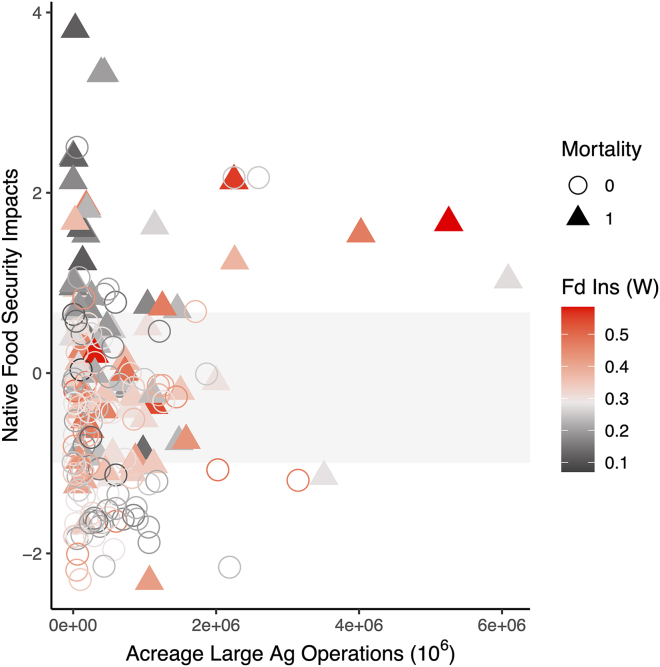
AI/AN pregnancy-related maternal death occurrence in relation to county acreage dedicated to large agricultural operations, county Native food security impacts, and county generalized Western food insecurity Filled triangles represent observation counties; open circles represent non-observation counties. Symbol color corresponds to county-level generalized Western food insecurity, with the midpoint between shades set at the median level of generalized Western food insecurity across all counties included in the analysis. Shaded horizontal bar represents the IQR for Native food security impacts (bottom: 1st quartile, top: 3rd quartile) across all counties included in the analysis. Native food security impact values above IQR represent very high impacts to Native food security; values below IQR represent very low impacts to Native food security.

Variables included in the most explanatory model reached Bonferroni multiple comparisons significance (*p* ≤ 0.004) for Native food security impacts (*p* < 0.001, Table 2), generalized Western food insecurity (*p* = 0.004, Table 2), the interaction between Native food security impacts and agricultural acreage dedicated to large agricultural operations (*p* = 0.001, Table 2), and the interaction between all 3 variables (*p* = 0.002, Table 2). AI/AN pregnancy-related maternal death occurrence was primarily driven by Native food security impacts, followed by generalized Western food insecurity, and then by the interaction between all 3 variables (Figure 3). AI/AN pregnancy-related maternal death occurrence separated above and below the IQR of county-level Native food security impacts. Counties with very high impacts to Native food security (>3^rd^ quartile, 0.672) were primarily those where mortality was observed, and counties with very low impacts to Native food security (<1^st^ quartile, −0.994) were primarily those where mortality was not observed (Figure 4). Patterns of generalized Western food insecurity were not associated with mortality at lower agricultural acreages; however, generalized Western food insecurity increased concurrently with county agricultural acreage (Figure 4). At very high county agricultural acreages (>3^rd^ quartile, >845,289 acres), counties with very low impacts to Native food security comprised approximately 1/5 of counties (21.569%), counties with high Western food security (>median, 0.287) comprised approximately 1/3 of counties (33.333%), and counties with both very low impacts to Native food security and high Western food security comprised approximately 1/10 of counties (11.764%) (Table S1). In all categories, higher food security was associated with counties that did not observe AI/AN pregnancy-related maternal death. The greatest disparities were present in counties with very low impacts to Native food security and in counties with both very low impacts to Native food security and high Western food security (Table S1).

**Table 2:** Model J

	Est	SE	t val	p-val
**Native food**	**1.144**	**0.213**	**5.382**	**1.720**^**e−**^**^7^**
**Western food (gen)**	**1.018**	**0.262**	**3.883**	**1.330**^**e−**^**^4^**
**Native food X ag acreage**	**−0.594**	**0.183**	**−3.251**	**0.001**
**Native food X Western food (gen) X ag acreage**	**0.780**	**0.249**	**3.132**	**0.002**
Native food X Western food (gen)	−0.451	0.279	−1.619	0.107
Western food (gen) X ag acreage	−0.443	0.307	−1.443	0.150
Ag acreage	0.065	0.230	0.281	0.779

Native food: county Native food security impacts; Western food (gen): county generalized Western food insecurity; ag acreage: county acreage dedicated to large agricultural operations; Est: estimate; SE: standard error; ag: agriculture; null deviance: 693.97 on 249 degrees of freedom; residual deviance: 517.80 on 242 degrees of freedom; bolded *p* values represent predictors meeting Bonferroni significance threshold (*p* value ≤ 0.004). Results for the model most explanatory of AI/AN pregnancy-related maternal death occurrence.

Because these data included mental health causes of death, we conducted a robustness analysis where records citing mental health-related causes of death were excluded to determine if this inclusion meaningfully impacted our results (Table S2). The same statistical protocols were then carried out on the reduced dataset. Variables included in Model J (Native food security impacts, generalized Western food insecurity, acreage dedicated to large agricultural operations) still produced the most informative model (Table S2), but only Native food security impacts reached statistical significance under the Bonferroni multiple comparisons significance threshold (*p* ≤ 0.004, Table S3). However, the main analysis and robustness analysis both showed that including Native food security, Western food insecurity, and agricultural acreage together yielded better model performances over those that only considered Native food security impatcs. Furthermore, the combination of variables outperformed Native food security impacts-only models by several ranks in both analyses. This consensus suggests that the explanatory power of Native food security requires a multivariate context.

### Agricultural intensity and climate (precipitation, temperature) are associated with changes to the nutritional status of coho Pacific salmon

**Figure 5:**
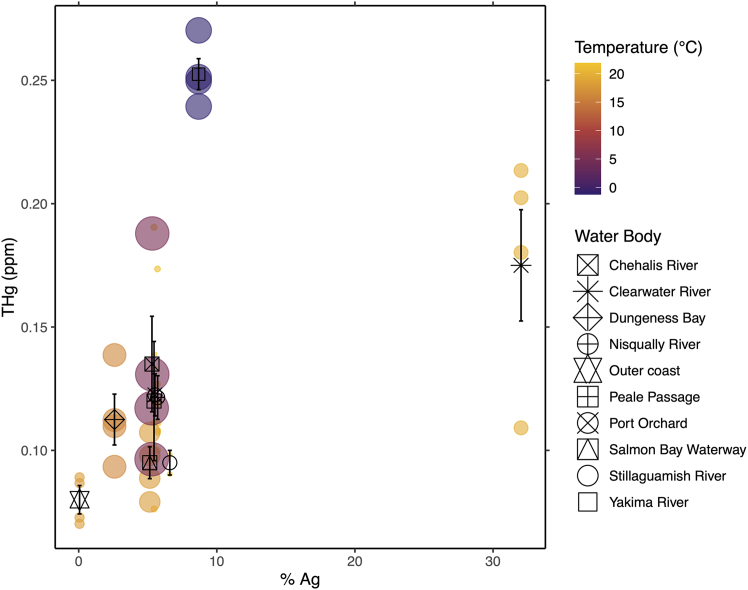
Total mercury Scatterplot shows the relationship between total mercury (THg) and agricultural intensity (% Ag), mediated by temperature and precipitation (climate). Temperature is the mean temperature of the previous month measured at the catch location (°C; min: −1.22°C, max: 21.86°C). Precipitation is the mean precipitation of the previous month measured at the catch location (inches; min: 0.08 in, max: 11.07 in). Colored bubbles represent individual samples. Color of bubbles represents temperature; size of bubbles represents precipitation. Black symbols represent the mean of total mercury for samples grouped by water body of origin; vertical error bars through black symbols represent the standard error of total mercury.

Total mercury (THg) was significantly explained by climate, agricultural intensity, and their interaction (Table 3). Fatty acid methyl esters (FAMEs) profile was significantly explained by agricultural intensity (Table 4). A nearly significant relationship between proximate nutrition (ash, moisture, crude protein, crude fat) and agricultural intensity was observed, but did not reach the Bonferroni multiple comparisons threshold enforced (*p* ≤ 0.017, Table 5). Levels of THg increased with agricultural intensity (Figure 5). Differences in interactions between climate variables (precipitation, temperature) and agricultural intensity were observed. Specifically, THg levels increased more in relation to agricultural intensity when fish were in climates characterized by higher precipitation and cooler temperatures. However, this study was not powered to test the statistical significance of this relationship.

**Table 3:** Total mercury (THg)

	Est	SE	t val	p-val
% Agriculture	0.008	0.001	7.450	7.180^e−9^
Climate	−0.047	0.012	−3.829	4.800^e−4^
% Agriculture x climate	0.010	0.002	5.703	1.590^e−6^

Multiple R-squared: 0.6987; adjusted R-squared: 0.6743; model *p* value: 9.608e^−10^; bolded *p* values represent predictors meeting Bonferroni significance threshold (*p* ≤ 0.017). Results for THg associations with agricultural intensity and climate.

**Table 4:** FAMEs profile

	Est	SE	t val	p-val
% Agriculture	0.223	0.074	3.021	0.005
Climate	0.347	0.838	−0.414	0.681
% Agriculture x climate	0.132	0.123	1.083	0.286

Multiple R-squared: 0.520; adjusted R-squared: 0.4811 model *p* value: 4.623^e−6^; bolded *p* values represent predictors meeting the Bonferroni significance threshold (*p* ≤ 0.017). Results for FAMEs profile associations with agricultural intensity and climate.

**Table 5:** Proximate nutrition profile

	Est	SE	t val	p-val
% Agriculture	0.139	0.059	2.359	0.024
Climate	−0.503	0.672	−0.749	0.459
% Agriculture x climate	0.144	0.098	1.460	0.152

Multiple R-squared: 0.2319; adjusted R-squared: 0.1696; model *p* value: 0.01952. Results for proximate nutrition profile associations with agricultural intensity and climate. Statistically significant effects were subject to a Bonferroni multiple comparisons significance threshold *p* value ≤0.017.

**Figure 6:**
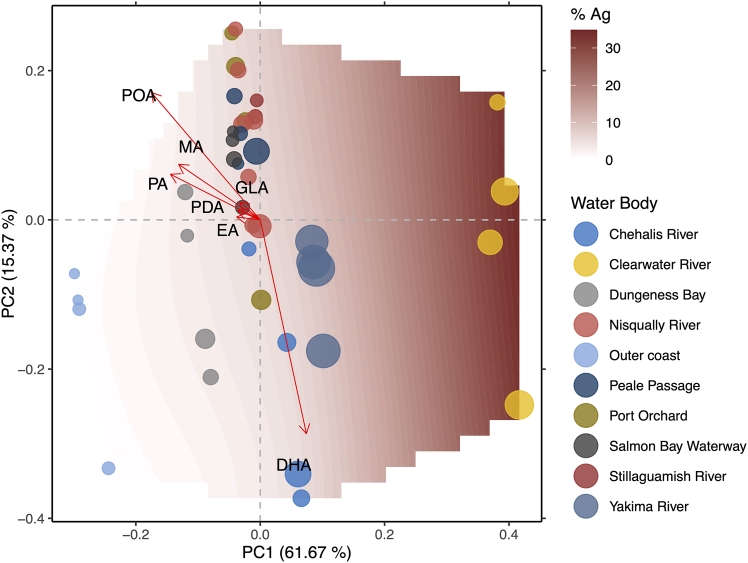
Fatty-acid methyl esters (FAMEs) Ordinal plot shows the relationship between sample-weighted FAMEs profiles and agricultural intensity (% agricultural land use). Contour surface represents agricultural intensity, with lighter shades of burgundy representing low levels of agricultural intensity that transition to deeper shades of burgundy with greater levels of agricultural intensity. Colored bubbles represent individual samples. Color of bubbles corresponds to the water body of origin, and the size of bubbles contextualizes the relative levels of total mercury measured in samples (parts per million; min: 0.07 ppm, max: 0.27 ppm). Vectors represent the magnitude and directional response of FAMEs, which significantly and strongly responded to agricultural intensity (r > 0.88, *p* < 0.05). Specific FAMEs corresponding to vector arrows are labeled in black. DHA: docosahexaenoic acid; EA: eicosadienoic acid; GLA: gamma-linolenic acid; MA: myristic acid; PDA: pentadecanoic acid; PA: palmitic acid; POA: palmitoleic acid.

FAMEs profile relationships were investigated by ordination (redundancy analysis, RDA). FAMEs most responsive to agricultural intensity were those at or exceeding the 3^rd^ quartile of r^2^ values for ordination goodness of fit (r^2^ ≥ 0.44). The most responsive FAMEs (*n* = 7) were docosahexaenoic acid (DHA), eicosadienoic acid (EA), gamma-linolenic acid (GLA), myristic acid (MA), pentadecanoic acid (PDA), palmitic acid (PA), and palmitoleic acid (POA). 6 of the 7 FAMEs (DHA, EA, GLA, MA, PDA, POA) have known or putative health benefits and 1 FAME (PA) imparts health risks in a maternal lipid-imbalance context (Table S4). With the exception of DHA, all eigenvectors for the most responsive FAMEs (EA, GLA, MA, PDA, PA) were primarily opposite to agricultural intensity increases along the PC1 axis, which explained most of the variation observed across FAMEs profiles (61.67%, Figure 6). The DHA eigenvector was approximately perpendicular to the 6 other FAMEs along the PC2 axis (15.37% variation explained, Figure 6), indicating that other factors in addition to agricultural intensity significantly influenced DHA levels and that there may be a tradeoff between levels of DHA and the levels of the other 6 FAMEs. Fish collected from the Clearwater River were associated with the highest agricultural intensity and had FAMEs profiles characterized by lower levels of EA, GLA, MA, PDA, and PA. Fish collected from the Yakima River, the Chehalis River, Dungeness Bay, and the outer coast, as well as select fish from other water bodies (Port Orchard, Clearwater River), were higher in DHA and lower in the other 6 FAMEs, and with levels of the other 6 FAMEs increasing in association with decreases in agricultural intensity. Profiles primarily characterized by higher levels of EA, GLA, MA, PDA, and PA were associated with fish collected from Puget Sound areas.

## Discussion

Maternal diet and nutrition at periconception and during pregnancy are leading modifiable risk factors in the development of poor pregnancy outcomes, including for AI/AN mothers.^35^ Also, the inability to attain optimal nutrition to support pregnancy is related to structural systems that determine vital components of human livelihood (i.e., social and structural determinants of health such as income, vehicle access, or proximity to medical services; and cultural determinants of health such as land, intellect, spirit, family, community). AI/AN Peoples experience disproportionate rates of Western food insecurity^36^ and poor pregnancy and maternal health outcomes,^4^ concurrently. When experiencing Western food insecurity, some individuals augment diets with traditional foods.^37^ We considered both Native and Western food security here, which are infrequently explored together quantitatively in academic literature and especially in the context of AI/AN pregnancy and maternal health disparities.

AI/AN pregnancy-related maternal death occurred most often during the postpartum phase (approximately 57% of records vs. approximately 38% during pregnancy). Causes of death were mainly represented by 4 categories: I00-I99 (diseases of the circulatory system); O00-O99 (pregnancy, childbirth, and the puerperium); S00-T98 (injury, poisoning, and certain other consequences of external causes); V01-Y98 (external causes of morbidity and mortality). Causes of death classified under the 11 other categories were relatively infrequently cited.

Mitigating contributing causes of death in the postpartum phase may provide a significant opportunity for intervention via leveraging AI/AN maternal diet and nutrition. The postpartum period, also referred to as the “fourth trimester,” is a major determinant of maternal mortality and morbidity. Crucial processes for healthy maternal recovery must occur in this phase, such as the rapid reversal of substantial cardiac remodeling that was necessary to support pregnancy.^38^ Approximately 85% of circulatory system causes of death cited in our data were specific to heart disease, and >20% of records cited circulatory system causes as contributors to postpartum death. This suggests that heart-healthy dietary interventions in AI/AN populations are particularly promising, regardless of focus on pregnancy. Osage Nation, for example, developed a gardening intervention in partnership with academic researchers (the Food Resource Equity and Sustainability for Health, “FRESH,” study), which observed clinical outcomes that were beneficial to heart health (decreased dietary carbohydrate, cholesterol, and caffeine)^39^ and could reasonably extend to pregnant and/or recently pregnant individuals.

Maternal diet modification is also increasingly investigated as a powerful strategy for improving maternal mental health during pregnancy^22^^,^^40^ and in the postpartum.^40^^,^^41^^,^^42^ In our data, approximately 40% of AI/AN pregnancy-related maternal death records cited mental health causes of death (substance use, suicide). While direct biological benefits of diet have linked maternal mental health benefits to higher intake of fish or PUFAs like omega-3 fatty acids,^43^ the unique role of traditional foods in cultural identity, community cohesion/thriving, and other psychosocial concepts within AI/AN (and other Indigenous) populations cannot be overlooked. Therefore, the potential of I/TFS to promote AI/AN maternal survivorship is significant and should be seriously explored as a solution. I/TFS should also be investigated in other contexts beyond death, such as AI/AN maternal wellbeing and morbidity.

I/TFS emphasizes revitalizing cultural relationships with the land and so encompasses land use practices and policies at multiple levels (individual, community, municipal, state, federal). We show that very high impacts to Native food security and high levels of Western food insecurity experienced by a county’s general population are associated with a greater occurrence of AI/AN pregnancy-related maternal death, and that agricultural land use influences this association. At very high agricultural acreages, both Native and Western food-secure counties are rare. Furthermore, Native food secure counties are especially skewed toward those that did not observe any AI/AN pregnancy-related maternal death. These data corroborate traditional knowledge systems that assert food security, and Native food security in particular, as protective in AI/AN pregnancy health.^44^ These data also suggest that the relationship between maternal diet and AI/AN pregnancy-related maternal death is exaggerated when mothers are living in association with high agricultural acreages.

Additionally, agricultural intensity and climate were dynamic predictors of the nutritional status of a key traditional food, coho Pacific salmon (*Oncorhynchus kisutch*). Quantities of the beneficial fatty acids EA, GLA, MA, PDA, and POA decreased in response to agricultural intensity. Quantities of DHA also decreased in response to agricultural intensity, but patterns of geographic distributions of DHA quantities in coho Pacific salmon in relation to the other most responsive fats suggest there may be tradeoffs between DHA content and the synthesis of other beneficial fats. Agricultural activity in our sampling region increases with distance from the Pacific Ocean. Pacific salmon cease eating upon commencing spawning migrations and so the distance Pacific salmon travel upriver will influence muscle tissue composition and energy reserves.^45^ Including this confounder as a weighting variable in our analyses allowed the examination of nutritional relationships with agriculture while enforcing an artificially equal distribution of distance from ocean across samples.^46^ DHA, EA, GLA, MA, PDA, and POA are essential for various functions that support neonate/infant growth and development (among others) and also have roles in oocyte maturation and maintaining maternal health during pregnancy. One fatty acid, PA, has been implicated as a risk to pregnancy health in lipid imbalance contexts, such as that in maternal obesity or when maternal diets are high in unhealthy fats. On the other hand, PA was associated with decreased risk of asthma development in children born to mothers with healthy diets. It is a necessary fatty acid for cellular membranes, and endogenously synthesized PA is a major component of both fetal adipose tissues and fatty acids in maternal milk in normal, healthy pregnancy (see references in Table S4).

Total mercury increased in response to agricultural intensity and climates characterized by cooler temperatures with higher precipitation. The relationship between total mercury levels with respect to agricultural intensity also appears to magnify with increased precipitation, although our study was not powered to statistically test this association. We did not determine sources of mercury present in coho Pacific salmon, but dietary mercury is understood to be the dominant route of exposure in fish.^47^ However, our results indicate an environmental exposure rather than a dietary exposure because mercury levels increased as distance from the ocean (i.e., assumed last food intake) increased. Aqueous mercury exposure occurs^47^ but the toxicokinetics of this route have not been studied in adult Pacific salmon (to our knowledge). In mosquito fish (*Gambusia affinis*) and redear sunfish (*Lepomis microlophus*), aqueous mercury is retained at rates higher than mercury removal rates, and the majority of dietary mercury is removed after 48 h while the majority of aqueous mercury is retained.^47^ Nevertheless, mercury retention and removal rates vary across species and are significantly influenced by multiple factors including water salinity, fish weight, and form of mercury.^48^ Relevant to the current study, it has been hypothesized that cold and wet climates in northern Europe promote the build-up of organic materials, including mercury, in soils associated with agriculture and adjacent lands.^49^ In this context, our data implicate settings associated with agricultural land as a constituent source of environmental mercury that migrating salmon are exposed to, with precipitation possibly functioning to enhance salmon exposure levels via increased transportation and deposition of mercury into agriculture-associated water bodies.

Since time immemorial, Pacific salmon species have been an important source of sustenance for Indigenous Peoples in Northern Pacific latitudes,^27^ including during pregnancy. However, a main concern of mercury contamination is exposure to methylmercury (MeHg), a component of total mercury that is neurotoxic and can cross the placenta to accumulate in the fetal brain.^50^ This has important cultural and health implications for AI/AN Peoples that have relationships with Pacific salmon as a traditional food, as many individuals will consume Pacific salmon frequently. For example, among Peoples of the Columbia River Basin (Pacific Northwest, United States), a typical Tribal member consumes an average of 16 servings of Pacific salmon per month, with some Tribal members consuming nearly 60 servings per month (compared to an average of 3 servings per month in the general United States population).^51^

There is a growing body of evidence supporting the conclusion that the impacts of MeHg on fetal and infant development are far outweighed by the high nutritional benefits of consuming fish and other seafood (e.g., PUFAs). For example, results from the Seychelles Child Development Study, which studied the effects of MeHg on fetal neurological development from mothers in the Republic of Seychelles found no consistent adverse effects of MeHg from high seafood consumption on offspring neurological development at 22 and 24 years of age and with 10 total evaluations through infancy to adulthood.^52^ However, consensus has yet to be reached regarding new evidence in fetal neurodevelopment and maternal MeHg exposure. MeHg was not specifically measured in these samples, but models for MeHg exposure during fish consumption by Li et al. (2024)^53^ showed lower MeHg concentrations in seafood from higher latitudes (compared to tropical latitudes), and that subsistence communities within United States territories (as an aggregated estimate of all subsistence communities) may consume >170g of seafood per day before exceeding MeHg EPA safe reference doses for chronic oral exposure (0.1 μg per kg BW per day^54^). It is unclear if levels of mercury measured in coho Pacific salmon here are of concern, but these estimates suggest that coho Pacific salmon are safe and beneficial for frequent human consumption.

Effect sizes of our best-performing model describing the occurrence of AI/AN pregnancy-related maternal death (Model J) indicate that Native food security in a multivariate context is a main variable informing this occurrence. Although the specific USDA food access research atlas variables used in the Native food security impacts variable will also impact Western food security, we did not seek to draw contrasts between the roles of Native and Western food security in AI/AN pregnancy health. Rather, we aimed to explore how known factors affecting access to (and achievement of) Native food security may be related to AI/AN maternal health outcomes. The appropriateness of this approach is supported by the absence of multicollinearity between the Native food security impacts variable and the Western food insecurity experienced by the general population of a county (Figure S1). The reduction in coho Pacific salmon fatty acids that are beneficial to pregnancy, as well as the increases in total mercury, associated with increases in agricultural intensity, support the need to consider the quality of traditional foods when present (in addition to availability). Indeed, inadequate food quality rather than food quantity was associated with increased prevalence of obesity, diabetes, and hypertension in an AI/AN study group living within Chickasaw and Choctaw Nation territories.^8^

This work is consistent with concepts present within many traditional and cultural knowledge systems, which link food, culture, and health. This is exemplified in a sentiment from an interviewed Indigenous leader, Sonia Quispe (Quechua Peoples, Rosaspata community, Peruvian Andes), who expressed sadness when traditional foods did not grow because it signaled to her that *Pachamama* (Quechua name for the sacred space where all human and non-human relatives among land, water, animals, and plants exist) was unwell.^55^ Understanding that these links are present contributes to concepts that operationalize sustainability of food systems within traditional ecological knowledge bases via reciprocal relationships with environments (i.e., care for the land in perpetuity means the land cares for Peoples in perpetuity).^55^ It is understood within many Indigenous communities that Native food security depends on I/TFS,^8^^,^^13^^,^^14^^,^^56^ in part because it recognizes that traditional foods may not persist without Indigenous self-determination and the full participation of Indigenous Peoples in their cultural and religious relationships with the land, water, and their plant and animal relatives therein.^8^^,^^13^^,^^14^ Importantly, I/TFS is compatible with goals held broadly across various medical, health, and political agencies for improving Indigenous health disparities because, through a Western lens, I/TFS is analogous to a wrap-around framework that addresses the physical, spiritual, relational, and mental wellbeing of Indigenous Peoples by emphasizing access to, and relationships with, nutritionally healthy, culturally appropriate traditional foods, and connections to land and other elements held in sacred relationship.

### Limitations of the study

A major limitation of this work is that known health risks associated with agricultural activity were not explicitly considered. Exposure to pesticides and/or other hazardous chemicals can harm human health in geographically and temporally heterogeneous ways. This can be influenced by size of^57^ or proximity to^57^^,^^58^ agricultural operations, structural factors such as indoor air^57^ or tap water^59^ quality, and environmental factors like air drift.^57^^,^^58^ In offspring, exposure to pesticides and other persistent agricultural chemicals have been linked to increased risk of birth defects^60^^,^^61^^,^^62^ and other adverse outcomes like preterm birth, small for gestational age, and low birth weight.^63^ In mothers, this has been linked to higher likelihood for developing hypertensive disorders of pregnancy^64^ and gestational diabetes mellitus.^65^ As such, environmental exposure to agricultural activities may compound impacts of reduced Native and Western food security in AI/AN pregnancy in highly variable ways. Future opportunities should be pursued to elucidate synergistic effects between diet as a modifiable risk factor and exposure to agricultural activities. This may be especially relevant to AI/AN pregnancy-related maternal death in light of the prevalence of circulatory and heart disease causes of death cited in the records analyzed here, and that both hypertensive disorders of pregnancy^66^^,^^67^ and gestational diabetes mellitus^68^ increase maternal risk of developing cardiovascular disease later in life.

A second limitation is that this work does not specifically evaluate the impacts of AI/AN community-driven food (and particularly, I/TFS) programs that may have been active within the counties analyzed. Tribally operated hatcheries that propagate culturally important fish species like Pacific salmon are prominent examples. Others include traditional knowledge dissemination classes held by the Spokane Tribal Network’s TFS initiative^69^; traditional foods preparation and distribution to Alaska Natives carried out by the Native Conservancy’s Indigenous Food Sovereignty program^70^; and bison restoration efforts across Northern Great Plains territories supported by the Intertribal Buffalo Council.^71^ Programs like these are only marginally considered in our analysis via the inclusion of AI/AN population counts, which makes significant assumptions about what the presence of AI/AN individuals, who may not be Indigenous to territories bounded within the specific counties, means for (Native) food security and AI/AN maternal health outcome. Considering the overarching relationships of Native food security to AI/AN maternal health detected in this study, we hypothesize that relationships between Native food security and maternal health will strengthen when these programs are appropriately integrated into future analyses.

Other limitations are that maternal characteristics were not considered. This was a consequence of study design, as our aim was to investigate broad patterns of food security and land use that may inform AI/AN pregnancy-related maternal death occurrence rather than roles for maternal characteristics that interact with a lived environment. Also, specific AI/AN Tribes and Nations to which individuals belonged, as well as resolutions finer than county-level, were not available. However, it is expected that factors influencing maternal health will change in severity and type across communities and geographies. Maternal characteristics such as maternal age, race, BMI, parity, medical insurance status, and others can have profound impacts on maternal health and pregnancy outcome as well. Future work would benefit from stratifying population risk based on interactions between maternal characteristics and county-level features.

We were also not able to account for all variables that may affect relationships identified in this work. For example, the March of Dimes maternity care desert database is a highly simplified summary of maternal care access (rating is based on the ratio of obstetric clinicians to births, the number of birthing facilities, and the proportion of women without health insurance^72^) and does not consider factors such as community support programs or social support networks. Also, several facilitators and barriers to Native food security could not be represented by USDA food access research atlas variables, such as state and federal land use policies,^29^^,^^30^ or the presence of partnerships between state and/or private entities and AI/AN-led groups that work to support Native food security.^29^ Finally, all confounders of coho salmon nutrition were not measured (e.g., fish sexual maturity), and specific agricultural activities were not compared (e.g., organic farms and specific crops). Therefore, the current study does not draw causative links or directionality between land use, Native and/or Western food securities, AI/AN maternal health outcomes, and traditional food nutritional quality.

A strength of our study is that we integrate established bodies of literature concerning ways that land use impacts both the natural environment and marginalized groups, with cultural and traditional knowledge systems that assert the interconnectedness of the natural environment with AI/AN health and cultural wellbeing. We also address areas of critical concern: AI/AN pregnancy-related maternal death and vital traditional foods. Our analyses of AI/AN pregnancy-related maternal death and the Coho Pacific salmon nutrition demonstrate that relationships exist between food security, land use, and AI/AN maternal health. This work provides support for the importance of programs that specifically promote Native (versus Western) food security. Furthermore, we identified a threshold of Native food security that may improve AI/AN maternal health outcomes and has potential health policy relevance. Future analysis of causality and direction of these relationships may be impactful for improving AI/AN maternal health disparities.

## Resource availability

### Lead contact

Requests for further information and resources should be directed to and will be fulfilled by the lead contact, Contessa Ricci, PhD (contessa.ricci@wsu.edu).

### Materials availability

This study did not generate new unique reagents or other materials.

### Data and code availability

• Data reported for the salmon nutrition analysis are available on GitHub: https://github.com/contessaricci/landuse-foodsecurity-AIANmatmort. Data for AI/AN pregnancy-related maternal death analysis are not published because this utilizes restricted data.
• Code for salmon nutrition analyses and figures (data pre-processing, dataset building, total mercury, proximate profile, FAMEs profile, and RDA) are available on GitHub: https://github.com/contessaricci/landuse-foodsecurity-AIANmatmort. Code for AI/AN pregnancy-related death analyses and figures (data pre-processing, dataset buiding, model building and comparison, ORs, robustness analysis) is shared by the lead contact upon reasonable request to comply with requirements to protect the confidentiality of individuals whose records were included in analyses.
• Any additional information required to reanalyze the data reported in this paper is available from the lead contact upon request.

## Acknowledgments

We thank the fishers and businesses that provided Pacific salmon samples: Robert Roose at the Stillaguamish Tribe of Indians Natural Resources, Jamie Gonzalez at Pacific Harvest, Aaron Brookes at the Jamestown S’Klallam Natural Resources Department Fisheries Division, Tony Forsman at Suquamish Seafoods, Tara Livingood-Schott at the Confederated Tribes of the Chehalis Reservation Natural Resources Department, Gregory Wolfe at the Yakama Nation Fisheries Mid-Columbia Coho Restoration Project, Craig Smith at the Nisqually Natural Resources Department Harvest Management Program, TK at the Kelly’s Fresh Fish, Scott Steltzner at the Squaxin Island Tribe Natural Resources Department, and Tad Iritani at Washington State University; we thank Mohammed R. Islam, Ph.D at Palouse Environmental Services for conducting the nutritional analysis; we thank our colleagues who volunteered their time to help process Pacific salmon samples: Shubhanker Sircar, Sabrina Haney, Bradford Dimos, Evan Barnes, Max Butensky, Tait Algayer, Tholen Blasko, Alyssa Williams; we thank Blake A. Foraker at the Washington State University Meat Lab for donating facility space and resources to process and store Pacific salmon samples; this research was supported in part by the 10.13039/100000002National Institutes of Health (5F32MD019202).

## Author contributions

Conceptualization, C.A.R.; data curation, C.A.R. and P.H.; formal analysis, C.A.R.; funding acquisition, M.P.; investigation, C.A.R., P.H., and A.L.; methodology, C.A.R., M.W.; project administration, C.A.R.; resources, C.A.R.; software, C.A.R., J.M.P.; supervision, C.A.R., M.W., L.P., A.E.O., J.M.P., and L.E.H.; validation, C.A.R.; visualization, C.A.R.; writing – original draft, C.A.R., A.E.O., and M.W.; writing – review and editing, C.A.R., M.W., A.E.O., P.H., M.P., J.M.P., and L.E.H.; author positionalities, Method S1.

## Declaration of interests

The authors declare no competing interests.

## STAR★Methods

### Key resources table

**Table:** 

REAGENT or RESOURCE	SOURCE	IDENTIFIER
**Biological samples**		

Tissue samples, coho Pacific salmon (*Oncorhynchus kisutch*)	This paper; Indigenous and localized fishers across a Pacific Northwest region	N/A

**Deposited data**		

Processed data and original code: coho Pacific salmon nutritional analyses and figures (data pre-processing, dataset building, total mercury, proximate profile, FAMEs profile, RDA)	GitHub: http://github.com/contessaricci/landuse-foodsecurity-AIANmatmort	N/A
AI/AN pregnancy-related maternal mortality records (data years 2016–2019, 2022)	CDC: Multiple Cause Mortality restricted use files (permission for data use granted by CDC National Center for Health Statistics)	N/A
Agricultural census data (2017, 2022)	USDA: National Agricultural Statistics Service (NASS)	N/A
Food security data	USDA: Economic Research Service	N/A
Land use map	USGS, MRLC Project: National Land Cover Database (NLCD)(CONUS)	N/A
Temperature map	NASA Earth Observations (*NEO*): LAND SURFACE TEMPERATURE [DAY] (1 MONTH - TERRA/MODIS)	N/A
Precipitation map	NWS, NOAA: Quantitative Precipitation Estimate (QPE)	N/A

**Software and algorithms**		

Statistical software	R Core Team	N/A
GIS software	QGIS	N/A

### Experimental model and study participant details

#### Coho Pacific salmon tissue samples

A total of 44 tissue samples from adult coho Pacific salmon (*Oncorhynchus kisutch*) migrating to spawning grounds during the 2022 fall run were collected for our case study. Samples were caught between October and November 2022 from 10 distinct water bodies across a Pacific Northwest region: Chehalis River, Clearwater River, Dungeness Bay (Pacific Ocean, near shore), Nisqually River (Puget Sound), the outer coast (Pacific Ocean, open water), Peale Passage (Puget Sound), Port Orchard (Puget Sound), Salmon Bay Waterway (Puget Sound), Stillaguamish River, and Yakima River (Figure 1). Catch locations spanned primarily across Washington, United States (*n* = 40), with samples from the Clearwater River collected in Idaho, United States (*n* = 4). GPS coordinates of catch locations were recorded for GIS analysis. All samples of Coho Pacific salmon were obtained from Indigenous fishers or Indigenous-owned businesses except for Clearwater River samples, which were obtained from a non-Indigenous local fisher.

Fishers and businesses provided 4 fish technical replicates per catch location (2 male, 2 female fish). Coho Pacific salmon samples were obtained from: Stillaguamish Tribe of Indians (*n* = 4), Muckleshoot Indian Tribe (*n* = 4), Jamestown S'Klallam Tribe (*n* = 4), Confederated Tribes of the Chehalis (*n* = 4), Yakama Nation (*n* = 4), Nisqually Indian Tribe (2 locations, *n* = 8), Squaxin Island Tribe (*n* = 4), and Suquamish Tribe (*n* = 4). We additionally obtained fish from an Indigenous-owned (Tsm’syen First Nations) business caught in Makah Tribe territory (*n* = 4), and fish from the local, non-Indigenous fisher caught in Nez Perce Tribe territory (*n* = 4). All fishers and businesses were compensated at asking price, with some choosing to donate samples for the study. All fishers and businesses recorded the sex of the fish and GPS coordinates of catch locations. Some fishers or businesses provided whole fish while others chose to provide a fillet only. If fishers or businesses provided a fillet only, the fork length, presence/absence of a hatchery mark (fin clip), and 5–10 scales were provided by the fishers. Fork length was used to infer fish weight in kg^73^ while scales were used to estimate fish age in yrs.^73^ If the fishers or businesses chose to provide whole fish, the information and scale samples were collected in house.

All fish samples were frozen at −20°C on day of catch. Samples that were shipped were shipped frozen overnight. All samples were stored long term at −80°C until muscle tissue collection for nutritional analyses. Because our sampling design relied on citizen fishers and businesses rather than trained researcher field sampling, a standardized estimation for fish sexual maturity could not be obtained. Although all samples at all geographic locations were caught while fillet quality was high and were also representative of fish that are consumed in AI/AN and other communities, sexual maturation can influence Pacific salmon nutritional status^74^^,^^75^ and we acknowledge this as a limitation of our sampling strategy.

### Method details

#### Data

##### Multiple cause mortality restricted use files

The Centers for Disease Control and Prevention (CDC) Multiple Cause Mortality restricted use files^76^ were used to identify AI/AN pregnancy-related maternal death. We include AI/AN pregnancy-related maternal death in data years 2016–2019, and 2022. Permission for restricted data use was granted by the CDC National Center for Health Statistics. Criteria to identify records of AI/AN pregnancy-related maternal deaths were: (i) biological female aged 18 years–55 years; (ii) ≥1 cause of death involving pregnancy, childbirth, and the puerperium; and/or obstetrical tetanus; and (iii) any combination of AI/AN ancestry. State and county associated with mortality records reflect state and county of residence.

Pregnancy-related maternal death was defined using International Classification of Diseases, 10th Revision (ICD10) codes indicating pregnancy, childbirth, and the puerperium (ICD10 O00-O99) and/or an ICD10 code indicating obstetrical tetanus (A34). Data years 2020 and 2021 were excluded due to changes in medical and maternal care that arose during the COVID-19 pandemic (e.g., complications exacerbated by hospital bed availability). Data years post-2022 were excluded due to changes in medical and maternal care resulting from the 2023 *Dobbs Decision* (e.g., geographic differences for receiving life-saving maternal emergency care^77^^,^^78^).

Records citing causes of death that were classified as vehicular, neoplasm, COVID-19 or respiratory infection (2022 records only), homicide, and exposure were excluded (*n* = 94). Records citing mental health-related causes (suicide, substance use) were included (*n* = 49) because there is an established relationship between pregnancy, mental health, and diet.^5^

##### USDA agricultural census

Large agricultural operations were defined as farm sizes ≥1,000 acres. Total county acreage dedicated to large agricultural operations was the sum of the USDA agricultural census bins for farm sizes 1,000–1,999 acres, and >2,000 acres). County-level acreage data were obtained using the 2017^79^ and 2022^80^ USDA agricultural census acreage estimates. Large agricultural operations were defined as farm sizes ≥1,000 acres. AI/AN pregnancy-related maternal deaths occurring in data years 2016–2019 were associated with agriculture acreage estimates in the 2017 agricultural census, while deaths occurring in data year 2022 were associated with estimates in the 2022 agricultural census. By default, non-observation counties were assigned acreage values using the 2017 agricultural census by default. Per state, a random subset of non-observation counties was assigned the 2022 agricultural census values to equal the number of AI/AN pregnancy-related maternal deaths recorded in that state in data year 2022. Year of agricultural census used for non-observation counties was also the year assigned for model weighting. Records from Alaska were excluded due to differences in agricultural census data collection and availability protocols (*n* = 10).

##### USDA food access research atlas (FARA)

Native food security impacts, generalized Western food insecurity, and AI/AN-specific Western food insecurity were defined using the USDA Food Access Research Atlas (FARA).^81^ The 2019 USDA FARA contains 148 variables relating to Western food security down to census tract resolution and is an update to the 2015 USDA FARA survey. Specific FARA variables used are listed in Table S5. FARA variable values are aggregated census tract data to the county-level.

We used the count from county AI/AN population determined to be at low food access (e.g., grocery stores, supermarkets) at ≥0.5 mi for county AI/AN-specific Western food insecurity, and we used the count of county general population determined to be at low income and low food access at ≥0.5 mi for county generalized Western food insecurity (Table S5). Distance and economic barriers to food access are established proxies for quantifying food security in academic literature^82^^,^^83^ and for public policy purposes.^84^ We apply >0.5 mi regardless of urban and rural residence because it has been shown that this can more accurately describe rates of Western food insecurity.^83^ It has also been shown that including vehicle insecurity as a criterion for Western food insecurity improves accuracy of Western food insecurity estimates,^83^ but we did not include vehicle insecurity in our definition. This was because vehicle insecurity was included in our summary variable for Native food security impacts (details below). While there are many studies that also exclude vehicle insecurity in Western food insecurity estimates, we acknowledge this as a limitation.

##### AI/AN maternal death weighting variables

County land area (mi^2^) was obtained from the United States Census Bureau QuickFacts query tool.^85^ County maternal care access rating was obtained from the March of Dimes Maternal Care Desert PeriStats query tool.^72^ State count of live births by mothers of AI/AN ancestry was obtained using the CDC Wide-ranging ONline Data for Epidemiologic Research (WONDER) query tool.^86^

##### GIS maps

Environmental data associated with coho Pacific salmon (*Oncorhynchus kisutch*) catch locations and dates were temperature, precipitation, agricultural intensity, and urbanization. Temperature was the month average of daytime temperatures (degrees Celsius, °C) and obtained from the month Land Surface Temperature map from NASA Earth Observations (NEO).^87^ Precipitation was the month average of the 24 h daily precipitation total (inches, in), obtained from the National Weather Service month to date maps.^88^ Urbanization was obtained from the Multi-Resolution Land Characteristics (MRLC) consortium 2019 Percent Developed Imperviousness map.^89^ Agricultural intensity was obtained from the MRLC National Land Cover Database map^90^ and was the sum of pixels classified as “Plant/cultivated” (i.e., pasture/Hay, cultivated crops).

#### GIS analysis

Environmental data associated with coho Pacific salmon (*Oncorhynchus kisutch*) catch locations and dates were obtained via analysis in QGIS software^91^ (version 3.34). Distance from ocean was estimated as river miles (miles traveled along a river body) from a river’s estuary (where a river mouth meets the Pacific Ocean) to the catch location by tracing the most direct path along river tracts.

Climate variables (temperature, precipitation), and agricultural intensity (% planted/cultivated) were quantified within a 15 mi radius centered on catch location coordinates. The 15 mi radius for our buffer zones was selected to account for the highly mobile nature of coho Pacific salmon during spawning migrations to breeding grounds, while also avoiding high overlap of catch location buffer zones. Climate and agricultural intensity values were calculated using the zonal statistics plugin,^92^ with the respective variable map as the raster layer and the buffer zones as the polygon layer.

To calculate our climate variables, temperature and precipitation measurements were averaged for the month prior to catch date to account for sampling bias (e.g., collecting during favorable weather conditions) as well as recent accumulated climate conditions that may influence nutritional characteristics. Because temperature and precipitation maps were by default averaged over calendar month (versus a rolling average of 24h days), samples collected in the first half of a month were associated with average temperature and precipitation values for the month previous to catch, while sample collected in the second half of a month were associated with values for the concurrent month of catch.

Urbanization was initially considered for coho Pacific salmon nutritional analyses and was defined as % impermeable surface within the 15 mi buffer zone. However, due to moderate multicollinearity between urbanization and climate variables (Figure S2) which remained after calculating our “climate” summary variable (Figure S3, details below), urbanization was excluded from further consideration.

#### Coho Pacific salmon nutritional analysis

Nutritional analysis was performed by Palouse Environmental Services (Pullman, WA, United States) on muscle tissues collected from coho Pacific salmon samples. Nutritional analysis included a proximate nutrition analysis (ash, moisture, crude protein, crude fat), a fatty acid methyl esters (FAMEs) profile (35 fatty acids measured), and total mercury (THg).

Because our sampling strategy did not allow collection of muscle tissue upon catch and prior to freezing, all fish were first thawed at 4°C overnight before muscle tissue collection. Measuring nutritional profiles from fish at this stage, however, is an accurate reflection of the nutritional status of coho Pacific salmon when AI/AN and other communities would consume these fish. After collection, muscle tissues were re-frozen at −80°C and shipped overnight to Palouse Environmental Services labs. Proximate variables were reported as weight per 100 g of tissue. FAMEs were reported as weight per crude fat per 100 g of tissue. THg was reported as parts per million (ppm). We do not assess effects of heterogeneity in freezing and storage methods on nutritional analysis^93^ and we recognize this as a limitation of the work.

### Quantification and statistical analysis

#### Statistical approach

All statistical analyses were conducted in R software (version 4.4.1).^94^ Weighted multivariate linear models with Bonferroni multiple corrections for model significance were the primary tests in our main analysis (nationwide associations between distributions of agriculture, Native and Western food security, and the occurrence of AI/AN pregnancy-related maternal death, tested at the county level) and our case study (nutritional characteristics of coho Pacific salmon associated with agricultural intensity and climate across a Pacific Northwest region).

In our main analysis, we compared counties where a mortality was recorded (observation counties) to a random sample of counties where AI/AN pregnancy-related maternal death was not documented (non-observation counties) in a balanced experimental design. Model comparison was used to identify the most explanatory set of food access variables (selected *a priori*) related to AI/AN pregnancy-related maternal death. A total of 11 models were compared (Table 1). Goodness of fit was used to determine the most explanatory model, assessed by the Tjur’s R^2^ for binary outcomes, root mean squared error (RMSE), and percent correctly predicted samples (PCP). Our non-observation dataset was developed by randomly selecting non-observation counties within a given state with replacement until the sample size was equal to the number of AI/AN pregnancy-related maternal death records in that state (final dataset: 122 observation counties; 131 non-observation counties).

Variables selected *a priori* for relationship with AI/AN pregnancy-related maternal death were: total acreage dedicated to large agricultural operations; magnitude of potential impacts to Native food security; rate of Western food insecurity experienced by the general population (generalized Western food insecurity); and rate of Western food insecurity experienced specifically by AI/AN populations (AI/AN-specific Western food insecurity). Weighting variables were: year of death, county land area (mi^2^); county maternal care access rating; and state count of live births by mothers of AI/AN ancestry. The Bonferroni multiple corrections significance threshold enforced was *p* ≤ 0.004. Goodness of fit metrics were calculated using the Performance package.^95^

In our case study, 3 specific hypotheses for coho Pacific salmon nutritional quality were tested for relationships with climate and agricultural intensity (proximate profile, FAMEs profile, THg). The Bonferroni multiple comparisons significance threshold enforced was *p* ≤ 0.017. Weighting variables were: fish sex, fish weight, distance from ocean, urbanization, and the presence of a hatchery marking. We treated the proximate analysis and FAMEs profile as a univariate response variable by collapsing proximate nutrition variables and fatty acids (respectively) by into summary nutrition variables (details below). Statistically significant relationships for the summarized nutrition variables (proximate analysis, FAMEs profile) were investigated further via data visualization if detected. IPTW for analysis of AI/AN pregnancy-related maternal death represented probability of mortality. Coho Pacific salmon nutritional analysis alternatively used IPTW for multiple treatments^96^ and represented probability of belonging to true water body of origin.

#### Summary variables

All summary variables were derived by collapsing specific data using principal component analysis (PCA). Values of the summary variables were the PC1 eigenvalues.

#### Native food security impacts variable

The summary variable “Native food security impacts” was used in our maternal death analysis to understand factors impacting traditional food access as an aggregate. Collapsed FARA variables were those which reflected factors documented in academic literature from Indigenous authorship which can improve or hinder Native food security (see FARA variables and references listed in Table S6). Collapsed FARA variables were urbanization (proportion of census tracts designated as urban), median family income (averaged median family income across census tracts), AI/AN population count (sum of AI/AN population across census tracts), and vehicle insecurity (proportion of households across census tracts that do not have vehicle access) (Table S5). The discriminatory power of the Native food security impacts variable is demonstrated in Figure S4.

#### Climate variable

The summary variable “climate” was calculated for Pacific salmon nutritional analysis due high multicollinearity between temperature and precipitation present at catch locations (Figure S2). Temperature and precipitation measurements within each catch location buffer zone were the data which were collapsed.

#### Nutritional outcomes variables

The proximate analysis and FAMEs profiles for Pacific salmon nutrition analyses were calculated to investigate cumulative differences in groups of nutrition metrics. The proximate nutrition variable collapsed ash, moisture, crude protein and crude fat measurements per salmon tissue sample. Likewise, FAMEs profile nutrition variable collapsed measurements for 35 fatty acid methyl esters per salmon tissue sample.

#### Outlier removal and model weighting

Differences in the scale of model variables were addressed by applying the robust scaler.^97^ Multivariate outliers were detected and removed from both datasets using median absolute deviation (MAD).^98^

MAD for analysis of AI/AN pregnancy-related maternal death in relation to food security and agriculture used a cutoff at 4 standard deviations and was based on: outcome (observation, non-observation), large agricultural operations acreage, county land area, state count of live births by mothers of AI/AN ancestry, county urbanization, county averaged median family income, county AI/AN population count, generalized Western food insecurity, AI/AN-specific Western food insecurity, and vehicle insecurity. We applied the outlier identification cutoff at 4 standard deviations because applying the common practice of 3 standard deviations heavily skewed outlier identification toward mortality observations (>76% of total outliers identified at 3 standard deviations), which reduced the mortality sample size by approximately 10% (versus 3% for the no-observation sample size).

MAD for coho Pacific salmon nutritional analysis used a cutoff at 3 standard deviations for outlier determination and was based on: fish weight (kg), distance from ocean, urbanization, agricultural intensity, climate (temperature, precipitation), and proximate nutrition analysis. FAMEs profile was not included because FAMEs variables are a proportion of crude fat. Fish age was also not included because a large proportion of fish (*n* = 30, 68.18% of samples) were aged the same (3 years).

Linear models for AI/AN pregnancy-related maternal death and Pacific salmon nutrition were weighted using Inverse Probability of the Treatment Weighting (IPTW).^46^ IPTW for analysis of AI/AN pregnancy-related maternal death represented probability of mortality. Coho Pacific salmon nutritional analysis alternatively used IPTW for multiple treatments^96^ and represented probability of belonging to the true water body of origin. Weighting variables for AI/AN pregnancy-related maternal death were: year of death, county land area (mi^2^); county maternal care access rating; and state count of live births by mothers of AI/AN ancestry. Weighting variables for Pacific salmon nutrition were: fish sex, fish weight, distance from ocean, urbanization, and the presence of a hatchery marking.

#### Building maternal death models for comparison

Our 11 weighted generalized linear models (Table 1) were developed using a forward stepwise strategy to test maternal death relationships in univariate models (Native food security impacts, generalized Western food insecurity, AI/AN-specific Western food insecurity, acreage dedicated to large agricultural acreage) and then multivariate models thereafter (Table S7).

Elevated multicollinearity (Spearman’s ρ > 0.6, Figure S1) was detected between generalized Western food insecurity and AI/AN-specific Western food insecurity. Therefore, we did not include generalized Western food insecurity and AI/AN-specific Western food insecurity in the same model despite this being an acceptable level of multicollinearity.^99^ Additional weak to moderate multicollinearity was detected between several other independent variables but were included together in AI/AN pregnancy-related maternal death models because Spearman’s ρ are within acceptable margins^99^ (Figure S1).

#### Post-hoc investigation

##### Robustness analysis

To confirm that inclusion of mental health-related records in our AI/AN maternal death analysis did not meaningfully alter conclusions, we conducted a robustness analysis^100^^,^^101^ where records citing mental health-related causes of were death excluded. The non-observation dataset was adjusted for robustness analysis by randomly removing counties to equal the total number of observation counties where mortalities did not result from mental health-related causes.

##### Redundancy analysis

Redundancy analysis (RDA) was used to investigate statistically significant relationships detected between summarized Pacific salmon nutrition response variables (proximate analysis, FAMEs profile) and respective model covariates. RDA was conducted using the vegan package^102^ and the ggordiplots package^103^ in R. The most responsive nutritional metrics of the summary variable were considered those whose r^2^ values for ordination goodness of fit were at or exceeding the 3^rd^ quartile of RDA r^2^ interquartile range.

## Supplemental information

Document S1. Figures S1–S4, Tables S1–S7, and Method S1
