# Supplementary material for: Land use is associated with Indigenous maternal death occurrence through Native and Western food security
Source: iScience. 2026 Apr 30;29(6):115948. doi: 10.1016/j.isci.2026.115948 (PMC13196452; doi:10.1016/j.isci.2026.115948)
Supplement: Document S1. Figures S1–S4, Tables S1–S7, and Method S1 [file mmc1.pdf]

iScience, Volume *29*

Supplemental information

Land use is associated with Indigenous maternal death occurrence through Native and Western food security

C.A. Ricci, A.E. Orloff, M. Wynne, L. Pflueger, P. Hallos, A. Lopez, M. Phelps, J.M. Postma, and L.E. Hebert

SUPPLEMENTAL FIGURES

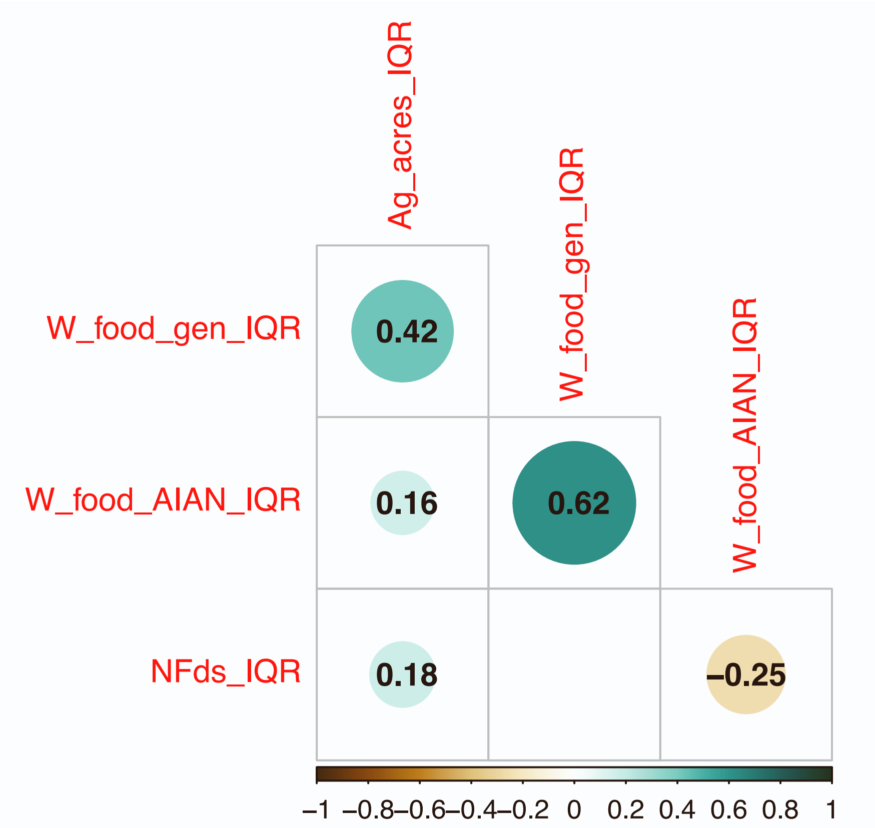

Figure S1 Correlation plot for model components included in AI/AN pregnancy-related maternal death analysis (related to STAR methods *Building maternal death models for comparison*). Variables are IQR normalized. Size of circle represents strength of correlation, color of circle represents direction of correlation, black numbers represent correlation coefficient (Spearman's ρ). Ag_acres_IQR: acreage dedicated to large agricultural operations; W_food_gen_IQR: generalized Western food insecuirty; W_food_AIAN_IQR: AI/AN-specific Western food insecurity; NFds_IQR: “Native food security impacts” summary variable

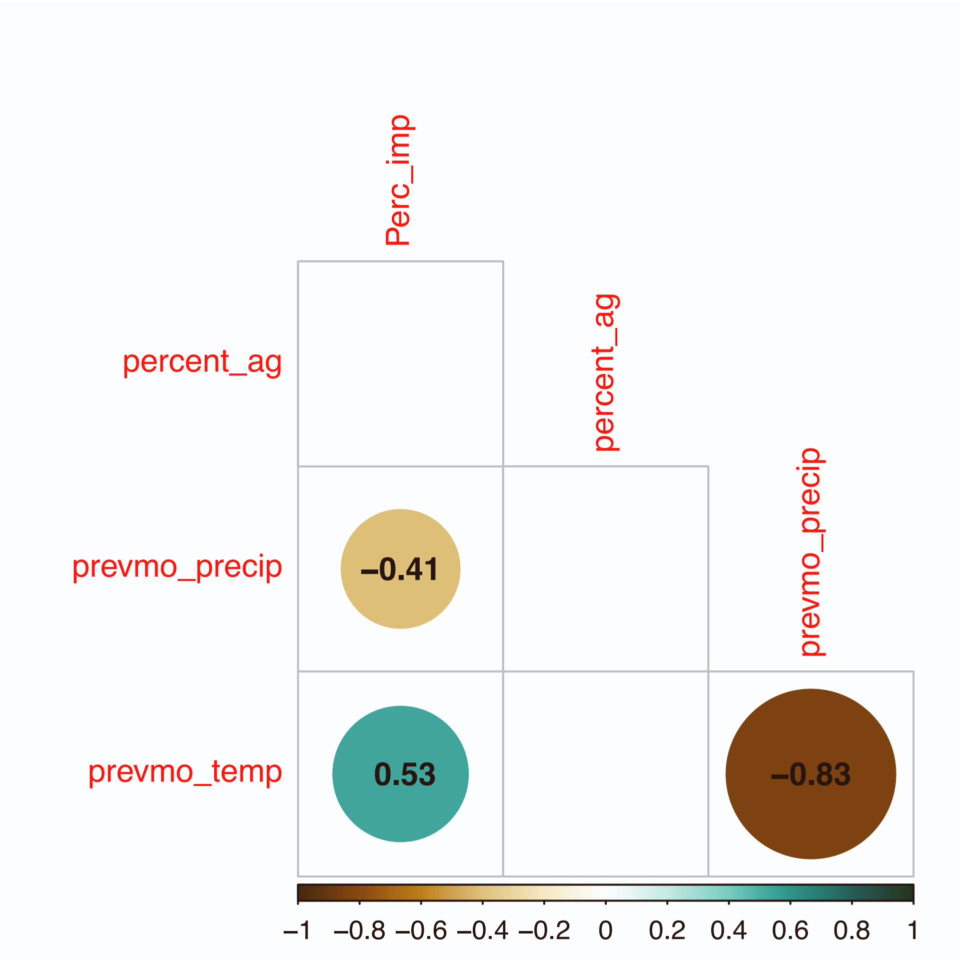

Figure S2 Correlation plot for multicollinearity between coho Pacific salmon model components for nutritional analysis prior to creation of climate variable (related to STAR methods *Summary variables*). Size of circle represents strength of correlation, color of circle represents direction of correlation, black numbers represent correlation coefficient (Spearman's ρ). Perc_imp: % urbanization; percent_ag: % agriculture intensity; prevmo_precip: average precipitation (in) for month previous to catch date; prevmo_temp: average temperature (°C) for month previous to catch date.

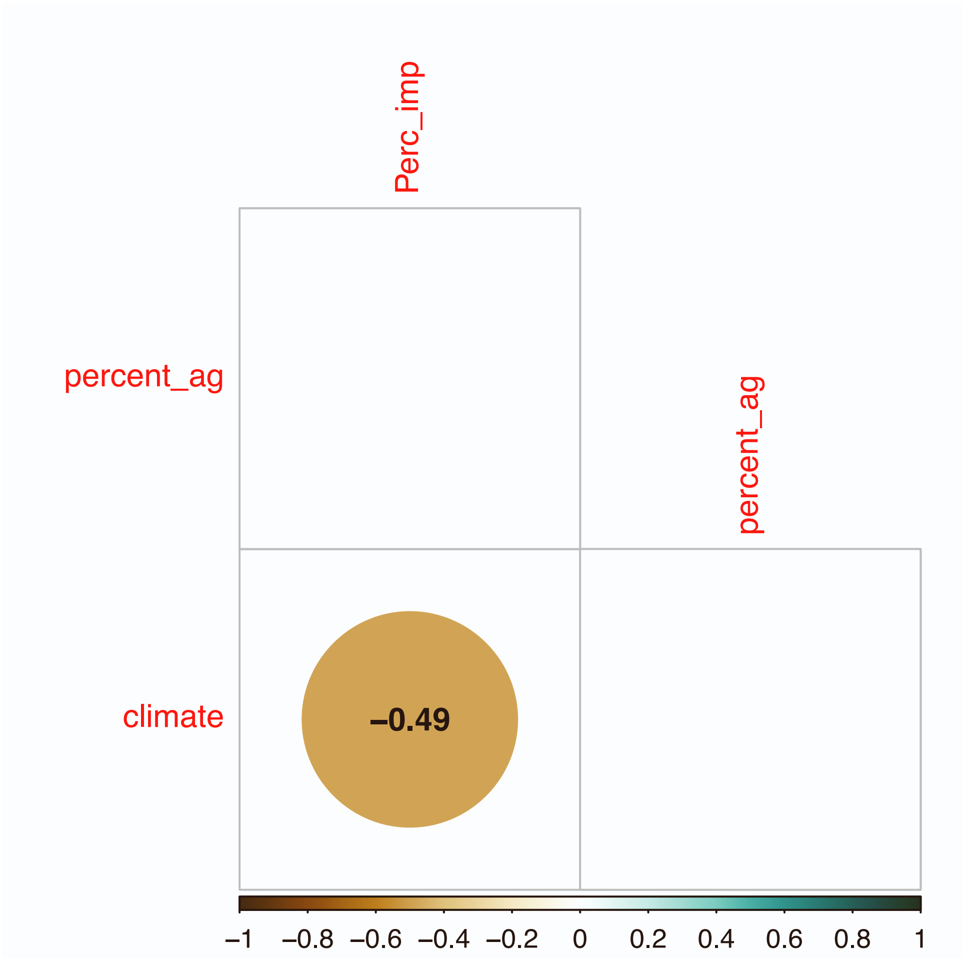

Figure S3 Correlation plot for model components included in coho Pacific salmon nutritional analysis after creation of climate variable (related to STAR methods *GIS analysis*). Size of circle represents strength of correlation, color of circle represents direction of correlation, black numbers represent correlation coefficient (Spearman's ρ). *Perc_imp: % urbanization;* percent_ag: % agriculture intensity; prevmo_precip: average precipitation (in) for month previous to catch date; prevmo_temp: average temperature (°C) for month previous to catch date.

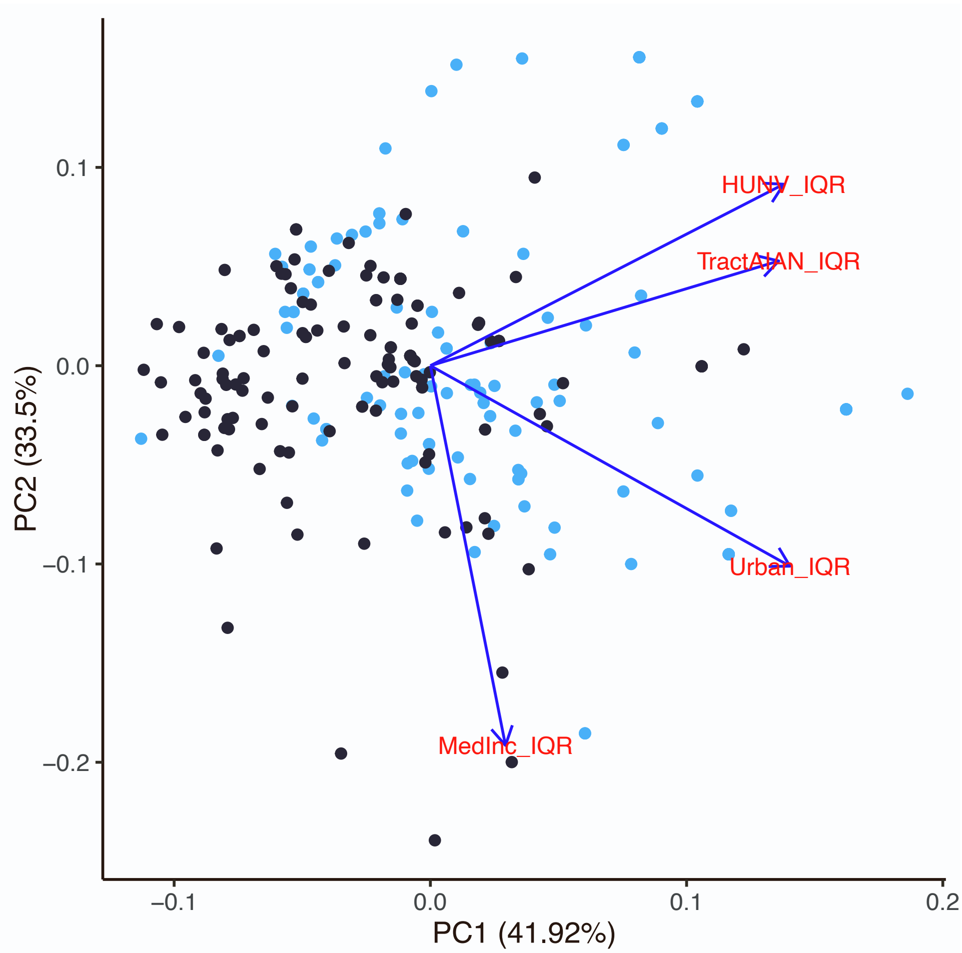

Figure S4 "Native food security impacts" summary variable (related to STAR methods *Summary variables*). PC loadings for IQR normalized USDA FARA variables included in "Native food security impacts" summary variable. Blue arrows: eigenvectors for variables included in summary variable. Black dots: counties where mortality was not observed; Blue dots: counties where mortality was observed. HUNV_IQR: generalized vehicle insecurity; TractAIAN_IQR: county AI/AN population count; Urban_IQR: proportion of census tracts within county that are designated urban; MedInc_IQR: median family income averaged across county census tracts.

SUPPLEMENTAL TABLES

Table S1 Food secure counties with very high agricultural acreage (related to figure 4). Pregnancy-related maternal death counts for non-observation vs. observation counties with very low Native food security impacts and/or high Western food security in association with very high agricultural acreage

**Very low Native food security impacts**		
Non-observation	Observation	Total
8	3	11
**High Western food security**		
Non-observation	Observation	Total
10	7	17
**Very low Native food security impacts and high Western food security**		
Non-observation	Observation	Total
5	1	6

Very high agricultural acreage: >3rd quartile (>845,289 acres); total counties with very high agricultural acreage: 51; non-observation counties with very high agricultural acreage: 24; observation counties with very high agricultural acreage: 27; very low Native food security impacts: <1st quartile (<-2.311); high Western food security: >median (>0. 287)

Table S2 Robustness analysis model comparisons (related to table 1). Model goodness of fit comparisons for county-level factors associated with AI/AN pregnancy-related maternal death occurrence. Models arranged in descending order from best fit to worst fit.

Model	Variables	Tjur's R^2	RMSE	PCP
RA_J	Native food, Western food (gen), ag acreage	0.301	0.421	0.651
RA_K	Native food, Western food (AI/AN), ag acreage	0.293	0.428	0.647
RA_E	Native food, Western food (gen)	0.234	0.436	0.617
RA_F	Native food, Western food (AI/AN)	0.222	0.438	0.611
RA_C	Native food	0. 216	0.436	0.608
RA_I	Native food, ag acreage	0.213	0.442	0.607
RA_H	Western food (AI/AN), ag acreage	0.140	0.471	0.570
RA_G	Western food (gen), ag acreage	0.038	0.480	0.542
RA_B	Western food (AI/AN)	0.008	0.497	0.504
RA_D	Ag acreage	0.001	0.501	0.500
RA_A	Western food (gen)	3.298^{e-4}	0.500	0.500

Native food: county Native food security impacts; Western food (gen): county generalized Western food insecurity; ag acreage: county acreage dedicated to large agricultural operations; RMSE: root mean squared error; PCP: % correct predicted

Table S3 Model RA_J (related to table 2). Results for the model most explanatory to AI/AN pregnancy-related maternal death occurrence in robustness analysis. Statistically significant effects were subject to a Bonferroni multiple comparisons threshold of p-value ≤ 0.004.

	Est	SE	t val	p-val
**Native food**	**1.190**	**0.388**	**3.064**	**0.003**
Western food (gen)	0.510	0.502	1.016	0.311
Ag acreage	-0.318	0.407	-0.782	0.436
Native food X Western food (gen)	-0.539	0.526	-1.026	0.307
Western food (gen) X ag acreage	0.375	0.553	0.678	0.499
Native food X ag acreage	-0.381	0.305	-1.247	0.214
Native food X Western food (gen) X ag acreage	0.473	0.395	1.196	0.234

Native food: county Native food security impacts; Western food (gen): county generalized Western food insecurity; ag acreage: county acreage dedicated to large agricultural operations; Est: estimate; SE: standard error; ag: agriculture; null deviance: 394.73 on 149 degrees of freedom; residual deviance: 282.51 on 142 degrees of freedom; bolded p-values represent predictors meeting Bonferroni significance threshold

Table S4 Health benefits and risks of FAMEs (related to figure 6). Examples of known or putative health benefits and risks of fatty acid methyl esters (FAMEs) in coho Pacific salmon that were most responsive to agricultural intensity, with a focus on health benefits during pregnancy and to offspring.

FAME	Health Benefit(s)	Reference
Docosahexaenoic Acid (DHA)	Infant brain development	Kuratko, Connye N et al. “The relationship of docosahexaenoic acid (DHA) with learning and behavior in healthy children: a review.” Nutrients vol. 5,7 2777-810. 19 Jul. 2013, doi:10.3390/nu5072777
	Infant cognition	Judge MP, Harel O, Lammi-Keefe CJ. Maternal consumption of a docosahexaenoic acid–containing functional food during pregnancy: benefit for infant performance on problem-solving but not on recognition memory tasks at age 9 mo2. The American Journal of Clinical Nutrition. 2007/06/01/ 2007;85(6):1572-1577. doi:https://doi.org/10.1093/ajcn/85.6.1572
Eicosadienoic Acid (EA)	Maternal gestational lipid metabolism	Li L-J, Lu R, Rawal S, et al. Maternal plasma phospholipid polyunsaturated fatty acids in early pregnancy and thyroid function throughout pregnancy: a longitudinal study. The American Journal of Clinical Nutrition. 2024/04/01/ 2024;119(4):1065-1074. doi:https://doi.org/10.1016/j.ajcnut.2024.02.016
	Offspring lipid balance	Vidakovic AJ, Jaddoe VWV, Voortman T, Demmelmair H, Koletzko B, Gaillard R. Maternal plasma polyunsaturated fatty acid levels during pregnancy and childhood lipid and insulin levels. Nutrition, Metabolism and Cardiovascular Diseases. 2017/01/01/ 2017;27(1):78-85. doi:https://doi.org/10.1016/j.numecd.2016.10.001
Gamma-Linolenic Acid (GLA)	Infant growth	Mychaleckyj JC, Zhang D, Nayak U, et al. Association of breast milk gamma-linolenic acid with infant anthropometric outcomes in urban, low-income Bangladeshi families: a prospective, birth cohort study. European Journal of Clinical Nutrition. 2020/05/01 2020;74(5):698-707. doi:10.1038/s41430-019-0498-6

	Infant cardiac development	Paredes A, Justo-Méndez R, Jiménez-Blasco D, et al. γ-Linolenic acid in maternal milk drives cardiac metabolic maturation. Nature. 2023/06/01 2023;618(7964):365-373. doi:10.1038/s41586-023-06068-7
Myristic Acid (MA)	Infant appetite	Gutiérrez-García AG, Contreras CM, Díaz-Marte C. Myristic acid in amniotic fluid produces appetitive responses in human newborns. Early Human Development. 2017/12/01/ 2017;115:32-37. doi:https://doi.org/10.1016/j.earlhumdev.2017.08.009
	Live birth	Kim K, Browne RW, Nobles CJ, et al. Associations Between Preconception Plasma Fatty Acids and Pregnancy Outcomes. Epidemiology. 2019;30
Pentadecanoic Acid (PDA)	Infant growth	Ciesielski V, Guerbette T, Fret L, et al. Dietary pentadecanoic acid supplementation at weaning in essential fatty acid-deficient rats shed light on the new family of odd-chain n-8 PUFAs. The Journal of Nutritional Biochemistry. 2025/03/01/ 2025;137:109814. doi:https://doi.org/10.1016/j.jnutbio.2024.109814
	Embryo development	Zarezadeh R, Nouri M, Hamdi K, Shaaker M, Mehdizadeh A, Darabi M. Fatty acids of follicular fluid phospholipids and triglycerides display distinct association with IVF outcomes. Reproductive BioMedicine Online. 2021/02/01/ 2021;42(2):301-309. doi:https://doi.org/10.1016/j.rbmo.2020.09.024
Palmitoleic Acid (POA)	Offspring lipid metabolism	Niinistö S, Takkinen H-M, Uusitalo L, et al. Maternal dietary fatty acid intake during pregnancy and the risk of preclinical and clinical type 1 diabetes in the offspring. British Journal of Nutrition. 2014;111(5):895-903. doi:10.1017/S0007114513003073
	Oocyte maturation	Mirabi P, Chaichi MJ, Esmaeilzadeh S, et al. The role of fatty acids on ICSI outcomes: a prospective cohort study. Lipids in Health and Disease. 2017/01/21 2017;16(1):18. doi:10.1186/s12944-016-0396-z

Fame	Health Risk(s)	Reference
Palmitic Acid (PA)	Placental inflammation	Shirasuna K, Takano H, Seno K, et al. Palmitic acid induces interleukin-1β secretion via NLRP3 inflammasomes and inflammatory responses through ROS production in human placental cells. Journal of Reproductive Immunology. 2016/08/01/ 2016;116:104-112. doi:https://doi.org/10.1016/j.jri.2016.06.001
	Offspring heart disease	Zhao R, Cao L, Gu W-J, et al. Gestational palmitic acid suppresses embryonic GATA-binding protein 4 signaling and causes congenital heart disease. Cell Reports Medicine. 2023;4(3)doi:10.1016/j.xcrm.2023.100953

Row colors delineate specific FAMEs and respective examples of health benefit(s) and risk(s)

Table S5 USDA FARA variables (related STAR Methods *Data*)Variables included in the 2019 USDA FARA survey that were used to calculate variables in analysis of AI/AN pregnancy-related maternal death occurrence. Table shows the main model component, the specific analytical variable, and the calculation using USDA FARA variables.

Model component	Analytical variable	Analytical variable calculation
Western food insecurity	AI/AN-specific Western food insecurity	Sum **laaianhalf** across county census tracts ÷ Sum **POP2010** across county census tracts
	Generalized Western food insecurity	Sum **lalowihalf** across county census tracts ÷ Sum **POP2010** across county census tracts
Native food security impacts	County urbanization	Sum **Urban** across county census tracts ÷ Total county census tracts
	Generalized low vehicle access	Sum **TractHUNV** across county census tracts ÷ Sum **OHU2010** across county census tracts
	County median family income	Sum **MedianFamilyIncome** across county census tracts ÷ Total county census tracts
	AI/AN population	Sum **TractAIAN** across county census tracts

Bold variables are names of specific variables included in 2019 USDA FARA that were used

Table S6. Barriers and facilitators to traditional food access (related to STAR Methods *Summary variables*). Factors identified from academic literature sources that were used to inform the “Native food security impacts” variable. Corresponding 2019 USDA FARA variables that were used in PCA are shown.

Barriers	FARA variable
Identity loss[a,b]<br>Knowledge loss[a,b,c,d]<br>Limited community support[a,b,d]<br>Lack of community programs[a,b]	*Total AI/AN population*
Costs of accessing traditional foods[a,b,c]<br>Cost of accessing knowledge[a]	*Median income*
Owning a vehicle[b,d]	*Vehicle insecure designation*
Urban residence[b,f]	*Urban designation*
Private land ownership[a,b]<br>Federal resource/land policies[a,b,d,f]<br>Land degradation[a,b,d,f]<br>Native foods contamination/quality[a,b,c,d,f]<br>Climate change[b,d,f]<br>Not enough Native foods[d,f]	
**Facilitators**	
Strength of cultural values[a,b,f]<br>Community engagement and activism[a,f]<br>Food sharing[b,f]<br>Sharing knowledge[b,f]	*Total AI/AN population*
External consultations and partnerships[a,f]	

Shaded cells are barrier/facilitator themes which can be represented by variables included in the USDA Food Access Research Atlas (FARA). Non-shaded cells are those which were not represented in “Native food security impacts” variable. Superscript letters correspond to referencing citing the barrier/facilitator.

References for supplemental Table S2:

a. Grann A, Carlsson L, Mansfield-Brown K. Barriers and supports to traditional food access in Mi’kma’ki (Nova Scotia) Canadian Food Studies/La Revue Canadienne des études sure l’alimentation. 2023;10:65-85. DOI: 10.15353/cfs-rcea.v10i1.571

b. Sowerwine J, Mucioki M, Sarna-Wojcicki D, Hillman L. Reframing food security by and for Native American communities: a case study among tribes in the Klamath River basin of Oregon and California. *Food Security*. 2019/06/01 2019;11(3):579-607. doi:10.1007/s12571-019-00925-y

c. Batal M, Chan HM, Fediuk K, et al. First Nations households living on-reserve experience food insecurity: prevalence and predictors among ninety-two First Nations communities across Canada. *Can J Public Health*. Jun 2021;112(Suppl 1):52-63. doi:10.17269/s41997-021-00491-x

d. Sowerwine, Jennifer et al. "Enhancing Indigenous Food Sovereignty and Community Health Through the Karuk Agroecosystem Resilience Initiative: We Are Caring for It: xúus nu'éethti." *Health promotion practice* vol. 24,6 (2023): 1096-1100. doi:10.1177/15248399231190368

e. Cidro J, Adekunle B, Peters E, Martens T. Beyond Food Security Understanding Access to Cultural Food for Urban Indigenous People in Winnipeg as Indigenous Food Sovereignty. Canadian Journal of Urban Research. 2015;24(1):24-43.

f. Blue Sky Minds Nonprofit, Washington State University, Council ORF. Okanogan Region Local Community Food System Assessment. 2024. Accessed 5/7/2025. https://static1.squarespace.com/static/642492ad34f4c9023a6143df/t/66ec8c514943b56429e62761/1726778452438/Okanogan-AssessmentBook_Sept2024_SinglePgs.pdf

Table S7 Description of models (related to STAR Methods *Building maternal death models for comparison*). Models compared in analysis of AI/AN pregnancy-related maternal death occurrence. Variables are aggregated at county level.

Model Level	Model	Variables
Univariate	A	Generalized Western food insecurity
	B	AI/AN-specific Western food insecurity
	C	Native food security impacts
	D	Agricultural acreage
2-variable multivariate	E	Native food security impacts * Generalized Western food insecurity
	F	Native food security impacts * AI/AN-specific Western food insecurity
	G	Generalized Western food insecurity * agricultural acreage
	H	AI/AN-specific Western food insecurity * agricultural acreage
	I	Native food security impacts * agricultural acreage
3-variable multivariate	J	Native food security impacts * Generalized Western food insecurity * agricultural acreage
	K	Native food security impacts * AI/AN-specific Western food insecurity * agricultural acreage

'' indicates full-factorial designation for model definition*

SUPPLEMENTAL METHODS

Method S1 Author positionalities (related to *Author contributions*).

CR is a cis Filipina and Italian woman who is an academic researcher at a land grant university (Washington State University) and is funded as an NIH fellow at time of publication (NIH 5F32MD019202). AO is cis Taiwanese American woman who is attending the University of British Columbia for her doctorate. She is studying Indigenous Resource Management in agroecological wetlands. MW is a citizen of the Spokane Tribe of Indians, an Interior Salish speaking people located on the Upper Columbia Plateau of what is currently called the USA. MW has a doctorate from the University of Hawaiʻi at Mānoa in Community and Cultural Concentration in Psychology and currently works with the Tribal Food Sovereignty and Indigenous Birth Justice teams at Spokane Tribal Network, a small non-profit on the Spokane Indian Reservation. LP is a cis woman who descends from the Spokane Tribe of Indians as well as German/Irish/English settlers and mother to a Hispanic (Mexican)/African American/Caucasian/Native American son. LP is a chef and currently works on the Tribal Food Sovereignty and Indigenous Birth Justice projects at the Spokane Tribal Network with her mother (MW). PH is a graduate of Washington State University currently working in aquaculture, providing health care to salmonid species for global food demand. AL is a Latino man who is a fish biologist for the Bureau of Reclamation in Yakima, Washington. AL is also a Ph.D. candidate at Washington State University in the School of Biological Sciences. MP is an Assistant Professor researching salmon physiology, conservation, and aquaculture. JP is a cisgender white woman who is a nurse and academic researcher at a land grant university (Washington State University). LH is a cisgender white women who is a public health scientist and academic researcher at a land grant university (Washington State University).
